# Cognitive remediation therapy for patients with eating disorders: a qualitative study

**DOI:** 10.1186/s40337-024-01101-0

**Published:** 2024-09-13

**Authors:** Tora Thorsrud, Marit Aspelund Bang, Camilla Lindvall Dahlgren, Trond Nordfjærn, Siri Weider

**Affiliations:** 1 Department of Psychology, The Norwegian University of Science and Technology (NTNU), Trondheim, Norway; 2 Eating Disorder Unit, Department of Psychiatry, Levanger Hospital, Nord-Trøndelag Hospital Trust, Levanger, Norway; 3 Division of Mental Health and Addiction, Vestfold Hospital Trust, Vestfold, Norway; 4 Department of Psychology, Oslo New University College, Oslo, Norway

**Keywords:** Transdiagnostic cognitive remediation therapy, Cognitive remediation therapy, Transdiagnostic treatment, Eating disorders, Cognitive difficulties, Qualitative

## Abstract

**Background:**

Transdiagnostic Cognitive Remediation Therapy (TCRT) is a new adaptation of cognitive remediation therapy for eating disorders (EDs) developed to address common cognitive difficulties across ED diagnoses (i.e., cognitive flexibility, central coherence, and impulsivity). This is the first evaluation of this novel treatment. The aim of this study was to explore acceptability and patients’ experience of TCRT.

**Methods:**

Thirteen patients diagnosed with restrictive or binge/purge subtypes of EDs and concurrent cognitive difficulties completed semi-structured qualitative interviews after receiving TCRT. Interview transcripts were analyzed using reflexive thematic analysis.

**Results:**

The analysis resulted in four main themes: (1) Treatment fit (2), Treatment experience (3), Perceived outcomes, and (4) Future recommendations. Eleven of the thirteen patients evaluated the treatment positively, found the focus relevant and expressed how it contributed to new insights related to thinking style. Seven of the patients also described it as a starting point for making changes and using new strategies. Importantly, experiencing some challenges related to the cognitive difficulties addressed in the treatment seemed essential for engagement.

**Conclusion:**

Offering TCRT as an adjunctive treatment for patients with EDs and concurrent cognitive difficulties can be a way to engage patients in treatment, build therapeutic alliances and provide important awareness and strategies to handle challenges related to thinking style.

**Trial registration:**

This study is part of a larger randomized controlled trial, ClinicalTrials.gov Id: NCT03808467.

**Supplementary Information:**

The online version contains supplementary material available at 10.1186/s40337-024-01101-0.

## Abstract (plain language summary)

Cognitive difficulties are thought to be one of several factors contributing to the development and maintenance of eating disorders (ED), but are rarely addressed in ED treatments. Cognitive remediation therapy (CRT) for EDs is a supplementary treatment originally developed for patients with anorexia nervosa (AN) that specifically targets cognitive difficulties. However, cognitive difficulties are found across ED diagnoses and not only in patients with AN. In this study, we have adapted CRT to address cognitive difficulties across ED diagnoses. The goal of this study was to explore patients’ experiences of this novel transdiagnostic CRT. We interviewed 13 individuals with various eating disorders after they had received the treatment. Eleven of the participants rated the treatment positively, expressed that it was engaging, offered something new and made them more aware of their thought processes. Seven participants also reported using new strategies to handle challenges related to cognitive difficulties. Importantly, one participant expressed that she did not experience cognitive difficulties and chose to drop out of the treatment, highlighting the importance of finding the treatment relevant to foster engagement.

**Supplementary Information:**

The online version contains supplementary material available at 10.1186/s40337-024-01101-0.

## Introduction

Eating disorders (ED) are severe and potentially life-threatening mental illnesses with dire consequences for physical health and psychosocial functioning [1]. Treatment of EDs shows unsatisfactory results and efforts are still needed to improve outcomes, as less than half of patients with EDs fully respond to treatment [2–4]. Additionally, long-term follow-up studies indicate that only 40–68% of patients with anorexia nervosa (AN) and 42–63% of those with bulimia nervosa (BN) achieve remission after 20 years or more [5–7]. Engaging patients in ED treatment can be challenging, as many patients feel ambivalence towards recovery, and treatment dropout is highly prevalent [8]. Although etiology is complex and multifactorial, neuropsychological functioning could be considered a contributing and maintaining factor for EDs [9, 10]. Consequently, addressing deficits in neuropsychological functioning, as well as treatment engagement, may be important to improve outcomes.

Several studies on neuropsychological function in EDs have been conducted the last decades. Despite some inconsistency in findings (e.g., one meta-analysis not finding evidence for set-shifting difficulties in BN [11], whereas another review reported no significant difference in set-shifting between AN, BN and binge eating disorder (BED) [12]) and methodological limitations, EDs have been found to be associated with a range of neuropsychological deficits including central coherence, cognitive flexibility, decision making and inhibitory control [13-15]

The primary research emphasis has been on AN, where numerous studies have shown difficulties in set-shifting or cognitive flexibility – the ability to alter behaviors or thoughts in response to contextual changes; and in central coherence – a processing bias favoring attention to detail at the expense of global processing [16, 17]. These difficulties have also been hypothesized to be precursors or endophenotypes of EDs [18, 19]. Recent meta-analyses have found difficulties related to set-shifting and central coherence in patients with BN [20, 21]. In addition, some studies have shown that individuals with BED struggle with set-shifting and impulsivity, indicating the need to address these difficulties in treatment [22–25].

Impulsivity is a multifaceted construct that is associated with deficient inhibitory control [26] A five facets model of impulsivity has been suggested in order to improve understanding of the impact of impulsivity on EDs and how different aspect of impulsivity could be related to different ED symptomatology [27]. These facets of impulsivity include tendencies to; engage in rash actions in response to negative emotions (negative urgency), engage in rash action in response to positive emotions (positive urgency), engage in behaviors with limited planning (lack of planning), pursue novel or exciting stimuli (sensation seeking), and limited capacity to maintain focus when distracted (lack of perseverance) [28]. Considering these facets could contribute to the understanding of common ED behaviors such as engaging in binge/purge behavior as response to negative emotions (negative urgency), disorganized eating patterns (lack of planning) or failure to maintain focus on weight restoration over time (lack of perseverance). As cognitive difficulties related to impulsivity may be present also in individuals with both AN and BN [29] as well as BED, addressing these issues related to impulsivity across EDs are important. Findings suggesting shared neuropsychological difficulties across diagnosis are in line with the transdiagnostic view which proposes possible shared contributing mechanisms across EDs [30]. Even though neuropsychological deficits are thought to be a predictor of treatment outcome [31], current evidence-based treatments do not target these mechanisms specifically [32].

Cognitive remediation therapy (CRT) for EDs, initially developed for AN, is an adjunct intervention developed to target inefficiencies in set-shift and central coherence [33]. CRT focuses on the process of thinking, rather than the content of thoughts. Through metacognitive exercises and behavioral tasks, the aim is to increase awareness of specific thinking styles and to develop adaptive strategies. The effect of CRT has been widely evaluated, mainly in AN, with promising results [34–38]. Given potentially shared contributing mechanisms and neuropsychological difficulties, it has been suggested that CRT might also benefit individuals with other ED diagnoses [20]. However, to date only two studies have evaluated CRT in patients with BN, BED and otherwise specified feeding and eating disorders (OSFED) [36, 39], finding CRT to contribute to reduction of ED psychopathology at follow up.

The majority of published studies on CRT for EDs have used quantitative methodology [38]. However, qualitative methods have become more widely used in psychotherapy research over the last decades, providing an important window into the complex processes involved in treatment of mental illnesses [40, 41]. Positive CRT evaluations originating from qualitative research have been documented in both adult and adolescent patients with AN, as well as in parents and clinicians [42–47]. In these studies, patients found CRT to be engaging, insightful into their thinking patterns, and beneficial in developing new cognitive and behavioral skills. Meanwhile, some patients also struggled to understand the treatment rationale and how it could help with their illness [42, 43, 47, 48]. Moreover, some patients expressed that performance anxiety could be challenging during treatment [49, 50]. These evaluations were based on written feedback letters or open-ended questionnaires, and although they provide important knowledge regarding the acceptability of CRT, in-depth interviews are imperative to understand how CRT is experienced by patients, and subsequently to use this knowledge to improve the treatment [48]. With the exception of one study published more than a decade ago [49], no qualitative CRT studies have been conducted for a transdiagnostic sample. The current study will add this important aspect to the literature.

Transdiagnostic CRT (TCRT) is an adaptation of CRT developed to address cognitive difficulties across ED diagnoses. The manual [51] was developed as a supplementary treatment and builds on existing CRT manuals for AN and obesity, as well as the CRT resource pack for adolescents [39, 52–55]. Exercises from these manuals were modified where needed to fit a transdiagnostic approach accommodating variation in cognitive challenges. Similar to other CRT manuals it addresses difficulties related to central coherence and set-shifting, but in addition exercises related to impulsivity have been added. This means that the focus in each session is not on diagnosis specific cognitive difficulties but rather if, and to what extent, patients experience challenges related to set-shifting, central coherence or impulsivity regardless of diagnosis. As in other CRT sessions, the goal in TCRT sessions was to identify patients’ cognitive styles, challenge ineffective thinking patterns and help the patients explore alternative ways of thinking, as well as promoting thinking about thinking (metacognitive awareness) using a variety of exercises and reflective questions.

The TCRT manual offers several optional modules, allowing the treatment to be tailored to each patient’s unique cognitive challenges. Treatment delivery is described in detail under Methods. An ongoing randomized controlled trial (Transdiagnostic Cognitive Remediation Therapy for Patient with Eating Disorders, a randomized controlled trial; TCRTRCT) is investigating the effect of TCRT as an adjunctive treatment in a transdiagnostic ED sample with co-occurring EDs and cognitive difficulties. The aim of the current study is to explore acceptability and patients*’ experience* of TCRT using in-depth semi-structured interviews in a sub-sample of patients with EDs from the RCT study consisting of patients with restrictive or binge-purge subtypes of EDs. This qualitative approach aims to enrich the evaluation of TCRT, which may shed light on the intricate processes influencing treatment outcomes.

## Methods

### Setting and recruitment

This study is an extension of the TCRTRCT study. Patients in treatment from the Regional Unit for Eating Disorders at Levanger Hospital or at the Eating Disorder Unit at St. Olav’s Hospital were invited. Both units are part of the public health care system in Norway and provide specialized eating disorder treatment at different levels of care (inpatient, outpatient or day-treatment) for patients with various level of illness severity and duration. Inclusion criteria for the TCRTRCT study were: meeting criteria for an ED diagnosis from the Diagnostic and Statistical manual of mental disorders, 5th edition (DSM-5), female sex, age 16–36 years, understand and speak Norwegian, being in treatment for an ED (inpatient, outpatient or day-treatment). Diagnoses were assessed by a clinical psychologist using the Norwegian translation of the Eating Disorder Assessment for DSM-5 (EDA-5) interview [56]. In addition, eligible participants were neuropsychologically screened and patients displaying cognitive difficulties were included in the study. Cognitive difficulties were defined as performing ≥ 1.0 standard deviation below the normative average in neuropsychological tests measuring set-shifting or central coherence, or self-reported cognitive difficulties on the Behavior Rating Inventory of Executive Function for Adults (BRIEF-A; [57]). After assessment, patients were randomized to either an active (treatment as usual (TAU) + CRT) or control condition (TAU). Patients in the control condition were offered TCRT after the 6-month follow up. For more details of the TCRTRCT study, visit ClinicalTrials.gov Id: NCT03808467.

Patients participating in the TCRTRCT study between May 2019 and February 2022 were invited to participate in the qualitative study by a member of the research team who had not been involved in their study participation or treatment. Interviews took place successively in the same time period. The inclusion criterion for the qualitative study were that the patients had completed at least 3 TCRT sessions, as this was deemed sufficient to form an impression of the treatment while at the same time making it possible to include patients who had prematurely dropped out of TCRT treatment. Including patients who had dropped out were deemed important to ensure a broad perspective on the treatment, thus giving an opportunity to enhance acceptability. Recruitment to the qualitative study was concluded after the given period, as an acceptable number of participants was reached.

### Measures

*The Eating Disorder Examination Questionnaire*, version 6 (EDE-Q, 58) is a self-report questionnaire developed to assess severity and frequency of core ED psychopathology the last 28 days. The EDE-Q is widely used in clinical and research settings and consist of 22 attitudinal items rated on a scale from 0 to 6 in addition to 6 behavioral items where patients report frequency of core ED behaviors (overeating behaviors and compensatory behaviors). Higher scores indicate higher severity of ED related symptoms. An optimal clinical cut-off score of 2.5 on the global EDE-Q score has been suggested in a Norwegian sample [59].

*The Behavior Rating Inventory of Executive Function- Adult version* (BRIEF-A, 57) is a self-report questionnaire assessing executive functions in everyday life. The BRIEF-A provides nine subscales related to different aspects of executive functions as well as two indexes; the Behavioral Regulation Index (BRI) and the Metacognitive Index (MI) and a summary score; Global Executive Composite (GEC). The BRI comprises the four subscales Inhibit, Shift, Emotional Control and Self-Monitor and the MI includes the subscales Initiate, Working Memory, Plan/Organize, Task Monitor and Organization of Material. Raw scores were converted to T-scores (M = 50, SD = 10). Higher scores reflect more problems related to executive functions.

### Treatment delivery

Patients received 9 weekly individual TCRT sessions in addition to TAU. Each TCRT session lasted approximately 45 min and was videotaped so that treatment fidelity could be assessed. The sessions consisted of different exercises designed to enhance metacognitive awareness and challenge ineffective thinking styles. Exercises included board games, pencil and paper tasks, puzzles, etc. Each exercise was followed by questions specifically designed to facilitate reflection on thinking processes, and alternative strategies challenging ineffective thinking patterns and making connections to everyday life. The therapist took a neutral stance, acknowledging the patients’ cognitive strengths and weaknesses while at the same time identifying whether cognitive styles were causing problems in patients’ everyday lives. As some patients could experience performance anxiety, the therapist emphasized the importance of concentrating on the thinking process and style, rather than on performance.

The first four sessions followed a set structure, each containing two exercises focusing on one of the cognitive styles (flexibility, central coherence, or impulsivity). In session five, an interim assessment allowed the therapist and patient to evaluate the treatment progress, and to identify exercises that the patient found particularly useful, and cognitive styles that were particularly challenging. Following the fifth session, the remaining sessions were tailored to best fit the patient’s needs. From here, exercises were chosen based on their relevance for the patient’s cognitive style, identified in the interim assessment. Throughout the treatment, patients also completed homework tasks between sessions. In the final session, the patient and therapist summarized key points from the treatment in writing.

### Data collection and analysis

Semi-structured qualitative interviews were conducted in person individually by TT, MAB, or SW. All three (TT, MAB, and SW) had also been involved in delivering TCRT in the current study, but interviews were always conducted by a research team member who had not been the patient’s TCRT therapist or part of the patients TAU treatment team. While utilizing a semi-structured interview guide (see Additional file 1), patients were actively encouraged to articulate their perspectives. Patients were informed that interviews would be anonymized and ensured that both positive and negative treatment experiences were equally valid and appreciated. The interview guide included questions regarding the patients’ ED, motivation for participating in the study, TCRT experiences and suggestions for TCRT improvements. The interviews lasted on average 32.5 min (SD = 12.3). All interviews were audio recorded and transcribed verbatim to text by TT and MAB. Transcripts were imported into Nvivo 20 for management and analysis. Transcripts were on average 11.8 pages (SD = 4.6) using font size 12.

Reflexive thematic analysis (RTA), as described by Braun and Clarke [60], was used as the framework when analyzing the data following their six-phased process. The method was chosen as it was appropriate to identify patterns of meaning across datasets, as well as emphasizing the importance of the researcher’s reflexive engagement with theory, data, and interpretation [61]. TT and SW approached the data with extensive knowledge of EDs, and clinical experience of delivering TCRT, in addition to several years of clinical experience with treating patients with EDs in general. Conversely, TN approached the data with no clinical experience with EDs, but some theoretical knowledge about TCRT and extensive knowledge of thematic analysis. These different viewpoints provided new insights and broader perspectives in the analytical process and were considered a strength.

An inductive and flexible approach was taken in the analysis, while at the same time recognizing that analysis in RTA is inescapably subjective [61]. TT listened to all interviews, in addition to reading all transcripts, to familiarize herself with the content before moving on to coding. Participants were given pseudonyms, and name of places, institutions, and other people were anonymized in the transcripts. Coding was mainly at the semantic level, but in later stages of coding some latent codes were also generated in the process of refining the codes. The codes where then used by TT as a basis for generating preliminary themes. TT, TN, and SW discussed, revised, and further developed themes until a final theme structure was agreed upon. Following the Braun and Clarke [62] checklist for quality, the themes were checked for internal homogeneity and external heterogeneity. Data saturation was not discussed, as the concept of saturation is not particularly suitable for RTA as it recognizes that different researchers generate different meanings during the analytic process [63].

### Ethics

The study was approved by the Regional Committee for Medical and Health Ethics of Central Norway (reference 2018/2418). All participants gave written informed consent. All patients who participated in the qualitative study were offered a debrief after the interviews, where participants were given the opportunity to express their experience of the interview and ask questions.

## Results

### Participants and treatment delivery

Thirteen patients were eligible for participation in the qualitative study during the given timeframe, and all of them consented to inclusion. Descriptive data is presented in Table 1. The diagnostic distribution was as follows: AN restrictive subtype (*n* = 4), AN binge/purge subtype (*N* = 5), BN (*n* = 2), and OSFED-atypical AN (*n* = 2). All patients were in voluntary treatment and deemed competent to make decisions regarding their treatment. Nine participants received inpatient TAU and four participants received outpatient TAU while receiving TCRT. Two patients dropped out of TCRT treatment prematurely. The reasons given for dropping out were not finding the treatment relevant (*n* = 1) and being discharged from the ED unit ahead of schedule, making it difficult to attend the sessions (*n* = 1). The decision was made to interview two patients who later completed all nine sessions of TCRT after session four as COVID-19 restrictions and lockdowns on the study sites posed a significant challenge to completing the TCRT treatment as well as having access to the patients. The average number of sessions completed when the interviews were conducted was 7.7 (SD = 2.3). All TCRT therapists were licensed clinical psychologists or advanced clinical psychologist students under supervision by a licensed clinical psychologist. All TCRT therapist had attended CRT workshops by Professor Kate Tchanturia or Professor Camilla Lindvall Dahlgren as part of their TCRT training. Only members of the research team had TCRT or CRT training during the study, thus TCRT techniques were not likely incorporated in the TAU treatment and were easily differentiated from the TAU treatment. Videos of one randomly selected session from each patient’s TCRT treatment course were assessed by the third author (CLD). The third author wrote a detailed assessment of each session, focusing on whether the TCRT therapist followed the structure and content of the specific session described in manual while at the same time assessing therapeutic alliance. Written feedback and evaluation were also provided to the TCRT therapist.”

**Table 1:** Descriptive data for the sample (*N* = 13)

	Mean	SD
Age (years)	23.9	4.4
Education (years) ^†^	13.8	2.4
BMI^†^	17.6	3.0
Duration of illness (years)^†^	8.7	4.0
Duration of ED treatment (years)^†^	6.4	4.0
EDE-Q		
Global score	4.41	1.1
BRIEF-A		
Inhibit	60.4	8.7
Shift	68.9	10.4
Emotional control	64.2	11.2
Self-monitor	52.5	13.5
Initiate	62.9	11.3
Working memory	71.5	10.7
Plan/organize	65.5	9.9
Task monitor	61.9	11.6
Organization of materials	49.2	14.0
BRI	64.8	9.9
MI	64.1	10.5
GEC	65.5	9.8

*Note* ED = Eating Disorder, EDE-Q = Eating Disorder Examination Questionnaire, BRIEF-A = Behavior Rating Inventory of Executive Function- Adult version, BRI = Behavioral Regulation Index, MI = Metacognitive Index, GEC = Global Executive Composite, ^†^Self-reported

### Qualitative findings

**Fig. 1:**
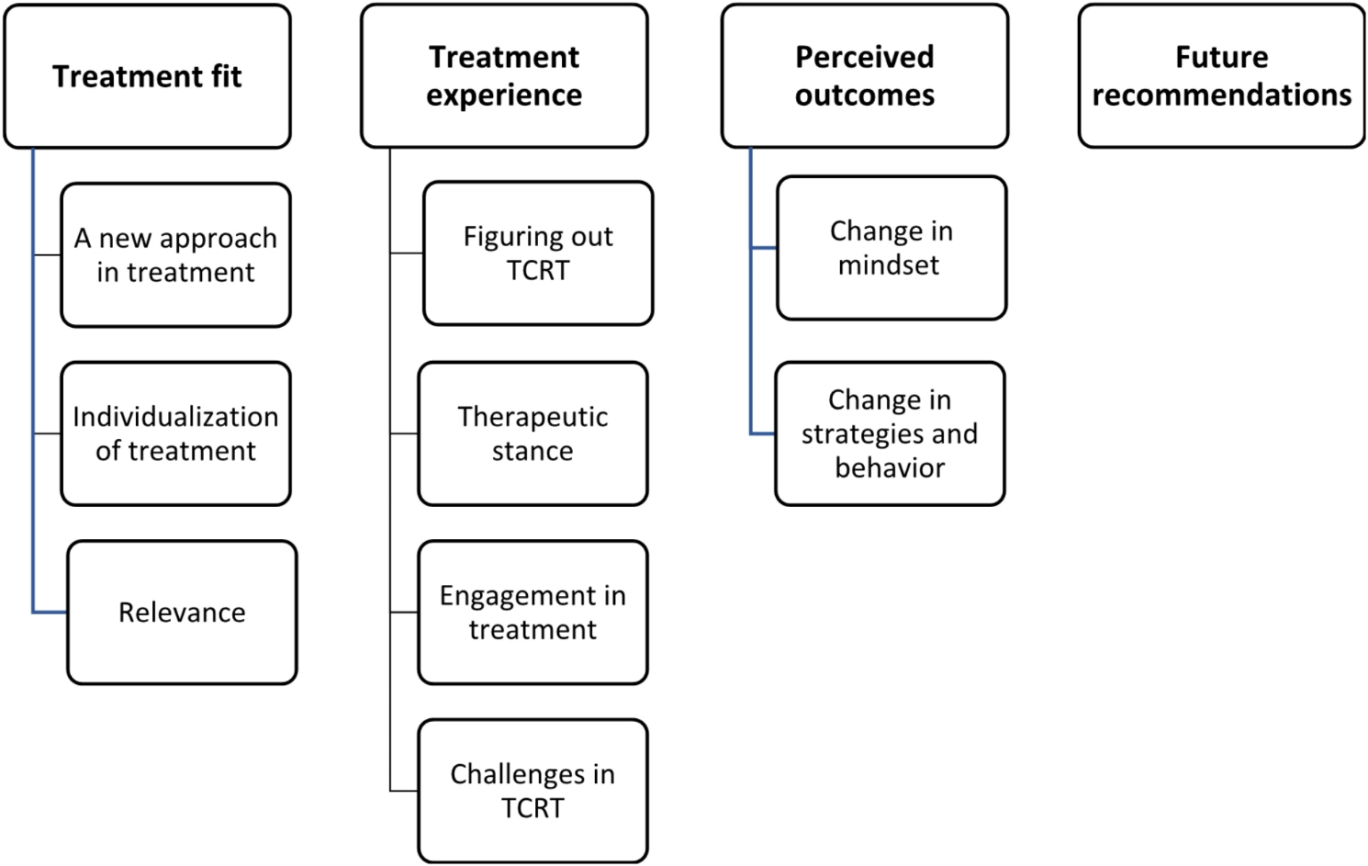
Themes and subthemes of patients’ experiences

The results of the qualitative analysis were four themes and nine subthemes. The structure of the themes and subthemes are presented in Fig. 1.

### Theme 1: treatment fit

Throughout the interviews patients described various ways in which the treatment matched their preferences and whether it addressed relevant topics concerning difficulties they were experiencing.

#### 1a A new approach in treatment

Seven of the patients mentioned how the approach in TCRT differed from what they had experienced in treatment before. The emphasis was on how TCRT was more suitable for them and their style of processing information, and that they welcomed the structure and concrete examples of the sessions. Six of the patients mentioned that they appreciated doing exercises and seeing concrete examples of thinking styles versus more traditional “talking therapies”. Fiona said: *“I feel like it’s a kind of approach that I haven’t done before. A way that might suit me better than ordinary outpatient treatment where you just talk because there is a lot of practical exercises.”* Four of the patients also noted that focusing on thought process rather than on content was beneficial for them and made it easier for them to communicate to the therapist about challenges they experienced. As Izzy described: *“I often find it hard to express what I’m thinking. So being in treatment is not very helpful*,* because my mind goes blank. But to rather think about how I’m thinking is easier than what I’m thinking about right now.”* She also related some of her challenges in therapy to difficulties recognizing and expressing emotions. The approach in TCRT seemed easier for her to benefit from and she went on to explain: *“I find it hard to know where to start and the emotional stuff have been very hard for me to understand*,* so I find this very okay. I believe that the emotional approach works well for many*,* and I’ve heard about people who show up for their session with a whole list of things that they want to talk about. And it’s not that I haven’t had a thought or emotion since the last session*,* I just can’t seem to get a hold of them.”*

#### 1b individualization of treatment

Six of the patients brought up that they appreciated the possibility of tailoring the treatment and that they found that this made the treatment feel more relevant for them. Being able to participate in the evaluation of treatment, as well as adjusting the course when needed, seemed to promote engagement and suitability of the treatment. As Hanna explained: *“We did have the evaluation on what we thought*,* or I thought*,* was useful and not*,* and then we adjusted the course to what I had a need for. And I thought that was very good.”*

#### 1c relevance

Eight of the patients confirmed that they experienced issues related to the thinking styles addressed and that focusing on these felt relevant to them. Danielle noted: *“When we talked about if I was detail oriented or focused on the bigger picture*,* I could come up with a lot of examples of when I see the details*,* like in everyday life.”* At the same time, four patients did not experience issues with all of the thinking styles addressed, and highlighted that having some sort of difficulties related to thinking styles was essential to finding the treatment relevant. Beth found none of the thinking styles relevant, and stated this as the reason for her dropping out of the treatment. *“I felt like it was not relevant to me*,* so it was like*,* I did not want to spend my time on it.”*

### Theme 2: treatment experience

The second important theme entailed different facets of their in-session experience. This was related to the processes of understanding the rationale of treatment that patients went through during treatment, as well as components of the experience they highlighted as important to them.

#### 2a figuring out TCRT

Initially, most patients found the structure and content of TCRT sessions unfamiliar. Eleven of them described going through a process during treatment from finding the approach strange to making sense of the treatment rationale, as well as figuring out how it could be applicable to their specific challenges. This process could take place within a single session for some, or throughout the course of treatment for others. Patients described reaching a turning point where they got a better understanding of the treatment, as well as viewing the purpose of previous sessions in a different light. As Gwen said: *“I didn’t quite understand what I’m supposed to do with this*,* for example those pictures. I was a bit like ‘what? I don’t know what this could say about what I am thinking’ (…). But then we get into a lot of interesting things (…) In the beginning I was thinking ‘this is weird’. And that I didn’t understand how it was useful. But then I’m left with a different impression at the end.”* Six of the patients highlighted the questions and reflections with the therapist as essential for their process of making sense of the treatment. Danielle explained: *“During the session I understand what we are doing*,* but if I had just shown up and was supposed to just do the exercises and then leave again*,* then I might not understand the purpose of it. It is because we talk about it that it makes sense.”*

#### 2b engagement in treatment

Overall, eleven of the thirteen patients described the treatment positively and that they were engaging in the treatment. Seven patients said they found learning about thinking styles interesting and described trying to apply the information to their own situations and challenges. Five patients also described the exercises as fun and enjoyable, and that engaging in the treatment was having a positive impact on them. Emily noted : “*It has been interesting to hear about the different thinking styles as well as doing the exercises.”*

#### 2c therapeutic stance

All patients said that they felt well taken care of by their therapist and that they had a good therapeutic alliance. Seven of them highlighted the efforts taken to reduce performance anxiety and how this was helpful for them during the treatment. Danielle, who struggled with performance anxiety, explained how the therapist’s focus helped her feel less anxious while doing the exercises: *“She (the therapist) does not care about the result. She cares more about how I do the exercises*,* and that makes me feel a lot less anxious.”*

Five of the patients also talked about how they experienced working together or collaborating with the therapist during TCRT, and how the therapist provided some new perspectives. They brought up how they appreciated that the therapist took a neutral stance regarding thinking style, giving them an opportunity to explore and evaluate what would be a useful approach for them moving forward rather than being told what they needed to change. Two patients also gave examples of previous negative experiences, where the therapist had told them what was best for them, or what they should do, without their opinion being heard. This was reflected by Mona, for instance, who noted: “W*hat I experience as useful is that I haven’t felt like there has been any moralizing and such. (….) there has not been anything like ‘your thinking is wrong’ or ‘you should think like this’ but more exploratory on what would be beneficial for me.”*

#### 2d challenges in TCRT

Several patients talked about challenges they experienced in the TCRT treatment. Five patients mentioned that they became focused on performance during the sessions and that they felt some degree of performance anxiety. These difficulties were not consistently connected to one specific exercise; rather, what patients found triggering could vary. Having issues with perfectionism and performance anxiety seemed to be an issue for some in general, and not just in TCRT sessions, however doing exercises with the therapist could also trigger an inner critic in the patients and lead to anxiety. As Anna explained: *“It often caused problems*,* the way I feel like I don’t do things well enough. It was something that I felt impacted me greatly during many sessions.”*

Eight of the patients also mentioned that the sessions could be demanding, especially if they were also in intensive inpatient treatment, as Emily pointed out: “S*ome of the exercises are demanding a lot of focus and thinking*,* (…) so when there is a lot going on during a day it could be a bit draining.”*

### Theme 3: perceived outcomes

#### 3a changes in mindset

Twelve of the thirteen patients described a new awareness concerning thinking style and how it could contribute to the difficulties they experienced. Kelly described: “*I experienced that I became a lot more aware of how I was thinking in different situations*,* not necessarily just during the tasks while I was there*,* but also out in everyday life.”* Importantly, eight of them also viewed the new awareness they had gained as a first step in making changes in their daily lives. As Anna explained: *“You have to start with becoming aware*,* (…) and then understand a bit more*,* and then start to think about how you can change.”*

#### 3b change in strategies and behavior

Although most patients described changes in awareness of thinking style, not all of them had started to make behavioral changes in their everyday lives. Seven of the patients mentioned starting to use new strategies developed in treatment. A recurring theme among those who described making changes was how they tried to challenge established rigid behavioral patterns they viewed as maladaptive or limiting. This extended beyond the session exercises and assigned homework to everyday situations. Some chose to challenge patterns related to food and exercise, while others chose other areas of their lives. Jenny described the effort she was making related to being more flexible: *“I make a little more active effort to do things differently. I’ve noticed that I’m getting better at it. I can often sort of challenge myself to do things differently on things that do not have anything to do with food and exercise and activity.”* Izzy explained how she now paused before acting, allowing her to assess her choices rather than acting on impulse: *“Earlier*,* I would just have jumped on the first idea*,* now I can think about if it usually pays off*,* or do I usually end up with a bad choice when I act on the first impulse.”* She went on to elaborate how the experiences gained through TCRT made her more open to being flexible: *“Like I said*,* the experience that I’m left with is that it’s actually fine to do things a little differently*,* and that’s something I’m taking away from this and will keep working on.”*

### Theme 4: future recommendations

Seven of the patients did not report any suggestions to improve the treatment. However, some gave valuable input on what could improve their experience. Three of the patients suggested to expand the treatment to more sessions or to have a booster session after some time. Two patients mentioned that it could be a good idea to create a patient workbook or an app that could include all the exercises, homework and reflections they made, making it easier for them to revisit previous sessions. Another patient also reflected on how some exercises seemed to be more suitable for patients in inpatient treatment and that she found some of the questions a bit repetitive.

## Discussion

The aim of this study was to explore acceptability and patients’ experience of TCRT. Four main themes were generated based on in-depth semi-structured qualitative interviews. Two of the themes were related directly to patients’ experience in sessions, with one focusing on perceived suitability of treatment and the other being linked to processes and mechanisms in treatment. The third main theme was related to perceived outcomes or changes as a result of the treatment. The last theme entailed suggestions for improving the treatment.

Patients’ descriptions of the treatment were generally positive. They engaged in the treatment and TCRT seemed to be well-tolerated. Seven of the patients also described behavioral changes and acquiring new strategies which they contributed to TCRT, in addition to gaining a new awareness regarding thinking style. These findings will be supplemented when the treatment outcomes measured by neuropsychological tests and assessments of ED symptomatology post-treatment for the TCRTRCT study are published. In line with previous research on CRT [44], TCRT also seemed to foster a good therapeutic alliance between therapist and patient, and taking a more neutral and exploratory stance, as well as collaborative efforts, were important factors. A contributing factor could also be that components of ED treatment that often put a strain on the therapeutic alliance, such as reducing underweight and limiting compensatory behaviors, are not the focus of TCRT. Even so, TCRT might be a good starting point when building a therapeutic alliance is challenging.

Patients expressed how they appreciated the focus on thinking styles in TCRT. At the same time, they all described the approach as unfamiliar or different from psychotherapeutic treatment they had encountered before. In line with findings from previous studies [47, 48], some patients initially struggled to understand the rationale and format of the treatment in our study. However, this was most apparent during the initial sessions, and eleven of the patients described overcoming these struggles during the course of treatment, which contributed to them viewing the treatment in a different light. These findings emphasize the importance of psychoeducation in treatment and of giving patients time to familiarize themselves with an unfamiliar treatment approach.

Eight of the patients described how TCRT addressed challenges relevant to them. In previous studies, some patients reported difficulties in seeing how CRT could help them with their challenges [42, 43, 48]. It is important to note that, in contrast to earlier studies, cognitive difficulties measured by neuropsychological tests or self-reports (BRIEF) were an inclusion criterion for the RCT study related to the current study. Thus, patients in the current study might have a higher prevalence of cognitive difficulties than the general ED population and would therefore find the treatment more relevant compared to patients with no or a lesser degree of cognitive difficulties. There is also a possibility that the individualization and tailoring of treatment based on the interim assessment helped make the treatment more relevant for patients. This suggests that CRT-based treatment might be better suited for patients with EDs and concurrent cognitive difficulties, and that this should be assessed before choosing TCRT as an adjunctive treatment. In addition, this highlights the importance of individualizing the treatment to fit each patient’s unique needs. Even though patients were screened for cognitive difficulties in the current study, one patient chose to drop out of treatment because she did not experience issues with thinking style as being relevant to her.

As in a previous study [47], patients also compared TCRT to other therapies, emphasizing what had not been useful for them before and what they experienced as helpful in TCRT. Interestingly, four of the patients noted that focusing on emotions or abstract concepts made it difficult to fully benefit from previous treatments. They argued that features of TCRT (focusing on concrete exercises or having a metacognitive focus rather than on thought content) made it easier for them to bring up relevant issues. Difficulties in recognizing and expressing emotions (alexithymia) is a problem for many patients with EDs [64]. Alexithymia has been suggested as a possible negative prognostic factor for treatment outcome for EDs [65]. Even though alexithymia was not assessed in the current study, the way some of patients described themselves in interviews seemed to be related to such difficulties. For them, TCRT might provide a stepping-stone to better communicate with their therapist about challenges they otherwise might struggle to express.

### Strengths and limitations

The study has several limitations. Inclusion of all types of EDs would have been preferable. Unfortunately, no patients with BED were eligible for inclusion during the data collection period in the qualitative study. This was also an issue for inclusion in the TCRTRCT study, where only one patient with BED was included. A probable reason is that patients with BED unfortunately seldom receive treatment at specialized ED units in Norway, but are rather treated at a different level of care (if at all). Future research on TCRT should take measures to ensure inclusion of patients with BED such as including study sites that provide treatment specifically for patients with BED. Another limitation is that member checking (participants giving feedback on transcript and analysis) was not performed throughout the study, and in future studies examining patient’s experiences of TCRT potential benefits of member checking should be considered.

A strength of this study is performing in-depth qualitative interviews, rather than data collection based on questionnaire or feedback letters, to get richer descriptions of patients’ experience of treatment. This also allows for a wider exploration of patients’ experiences and makes it possible to pursue relevant topics brought up during the interviews. Another strength was that the TCRT was delivered by different therapists, making it less likely that the patients’ feedback and experiences were therapist-dependent, but rather linked to the treatment itself.

## Conclusions

The current study is part of the evaluation of a new adaptation of TCRT development to address cognitive difficulties across ED diagnoses. It is the first study to explore patients’ experience of any version of CRT through in-depth interviews. The findings are in line with previous studies of CRT in general, but also provide important insights into patients’ experience of TCRT. Providing TCRT as an adjunctive treatment for patients with EDs and concurrent cognitive difficulties could be a way to engage patients in treatment and to build an alliance, while also providing important awareness related to thinking style and new strategies to handle challenges. It could also offer some patients an alternative gateway to discuss relevant topics in therapy. However, the outcomes of treatment in a larger group, as well as over time, still need to be evaluated.

## Electronic supplementary material

Below is the link to the electronic supplementary material.

Additional file 1

## Data Availability

The qualitative data analyzed in the current study are not openly available due to ethical/privacy restrictions as permission for data sharing has not been provided by participants or the Ethical Committee.

## Abbreviations

ED: Eating disorder
AN: Anorexia nervosa
BN: Bulimia nervosa
BED: Binge eating disorder
CRT: Cognitive remediation therapy
OSFED: Otherwise specified feeding and eating disorders
TCRT: Transdiagnostic cognitive remediation
TCRTRCT: Transdiagnostic Cognitive Remediation Therapy for Patient with Eating Disorders, a randomized controlled trial
DSM-V: Diagnostic and Statistical manual of mental disorders, 5th edition
EDA-5: Eating Disorder Assessment for DSM-5
BRIEF-A: Behavior Rating Inventory of Executive Function for Adults
TAU: Treatment as usual
RTA: Reflexive thematic analysis
