# Supplementary material for: Cognitive remediation therapy for patients with eating disorders: a qualitative study
Source: J Eat Disord. 2024 Sep 13;12:142. doi: 10.1186/s40337-024-01101-0 (PMC11401416; doi:10.1186/s40337-024-01101-0)
Supplement: Supplementary file 1 — Additional file 1 [file 40337_2024_1101_MOESM1_ESM.docx]

**Appendix 1**

**Interview Guide TCRT – English Translation**

Today, I would like to talk to you about your experiences participating in the study "Cognitive Training for Patients with Eating Disorders," where you engage in cognitive exercises, often referred to as brain training, as a part of your treatment. The questions I will be asking serve as a guide, and I am keen to hear about your personal experiences. You are welcome to share freely, and please remember that there are no right or wrong answers here. Before we begin, I want to emphasize that your participation is entirely voluntary, and that you are free to withdraw at any time. Should you need to take a break, please feel free to do so.

1. Could you share some insights into your illness journey?

- How long have you been dealing with eating disorders?
- What treatments have you tried previously?
  - What has been your experience with these treatments?
- What motivates you to engage in brain training?
- How did you learn about the study?
- What were your initial hopes regarding your participation in the study?
- What do you expect to achieve by participating?

1. How would you describe your experience with the brain training so far?

- Have you encountered any aspects that were effective or beneficial? Or something that did not seem useful?
- Have you noticed any changes in your own thought processes as a result of the training?
  - If yes, what kind of changes?
- Have you felt a sense of progress through the training?
- What are your thoughts on working on your thinking style?
- Did you feel adequately supported before, during, and after the training sessions?

1. Do you have any suggestions or thoughts on aspects of the training that could or should be altered?

- Were there elements of the training that you found to be ineffective?
- Are there aspects of the training that you believe could be modified?

*If the participant has discontinued the TCRT program*

1. What led you to discontinue the program before completion?
   - Did you find any aspect of the study to be too challenging?
     - Were the tasks too difficult? Was the training overly demanding? Was there an issue with the implementation of the training that did not work for you?
   - Did you experience a lack of progress?
   - In hindsight, is there anything that could have been done differently to enhance your brain training experience?

*Debriefing with all participants*

- How did you feel about the interview process?
- Were there any subjects that you found challenging to discuss?
- Is there anything you believe should have been addressed that I did not ask about?
- Now that the interview is complete, do you have any questions for me?
